# Identifying Emotional Expressions: Children’s Reasoning About Pretend Emotions of Sadness and Anger

**DOI:** 10.3389/fpsyg.2020.602385

**Published:** 2020-11-30

**Authors:** Elisabet Serrat, Anna Amadó, Carles Rostan, Beatriz Caparrós, Francesc Sidera

**Affiliations:** Department of Psychology, University of Girona, Girona, Spain

**Keywords:** sadness, anger, pretend emotions, children, emotional expression

## Abstract

This study aims to further understand children’s capacity to identify and reason about pretend emotions by analyzing which sources of information they take into account when interpreting emotions simulated in pretend play contexts. A total of 79 children aged 3 to 8 participated in the final sample of the study. They were divided into the young group (ages 3 to 5) and the older group (6 to 8). The children were administered a facial emotion recognition task, a pretend emotions task, and a non-verbal cognitive ability test. In the pretend emotions task, the children were asked whether the protagonist of silent videos, who was displaying pretend emotions (pretend anger and pretend sadness), was displaying a real or a pretend emotion, and to justify their answer. The results show significant differences in the children’s capacity to identify and justify pretend emotions according to age and type of emotion. The data suggest that young children recognize pretend sadness, but have more difficulty detecting pretend anger. In addition, children seem to find facial information more useful for the detection of pretend sadness than pretend anger, and they more often interpret the emotional expression of the characters in terms of pretend play. The present research presents new data about the recognition of negative emotional expressions of sadness and anger and the type of information children take into account to justify their interpretation of pretend emotions, which consists not only in emotional expression but also contextual information.

## Introduction

This study explores children’s capacity to comprehend that the emotions expressed in pretend play contexts may have playful intentions. This capacity to detect emotions simulated by other people is considered important because it helps people to identify reliable individuals and establish positive and trusting relationships with others, and to communicate effectively in social contexts (Saarni et al., 2007; Walle and Campos, 2014). Specifically, this research focuses on how the ability to discriminate facial expressions of emotion is developed, but with particular emphasis on the more specific ability of detecting pretend emotion (or emotions simulated in pretend play contexts), so as to understand how children explain their interpretations of pretend facial expressions. In addition, this emotional recognition is studied in the context of pretend play, where the simulation of emotions often occurs in childhood. Regarding this, it is assumed that contextual information is fundamental in the recognition of facial expressions and pretend emotions. We will now discuss these aspects of the study in more detail.

### Children’s Recognition of Emotional Expressions

Recognizing the emotions of other people through their facial expression is important in human relationships. It is an essential ability in interpersonal interactions, since it allows us to behave properly in different social contexts, is an important aspect of interpersonal communication, and is crucial in regulating the behavior of others (Saarni, 1999; Scharfe, 2000).

Throughout their development, children progress in their ability to identify and understand facial expressions associated with emotions. Babies begin to discriminate emotions in facial expressions during the first year of life (Morgan et al., 2010). Toward the end of the first year and at the beginning of the second, babies try to give meaning to situations based on the information obtained from the emotional expressions of others, a skill that has been called social reference (Sorce et al., 1985; Widen and Russell, 2008). Therefore, at this age children already understand that people’s emotions have a meaning linked to external events, and after 14 months they are able to identify where the emotion is directed (Repacholi, 1998), and to match some negative emotions to specific eliciting events (Ruba et al., 2019).

Developing the recognition of facial emotional expressions can be understood as a process of increasing expertise in the ability to discriminate emotions (Widen, 2013). According to some authors the ability to recognize basic emotional expressions could begin with the distinction between two broad categories - feel good, feel bad (Widen and Russell, 2010; Widen, 2013) - and improves throughout childhood and adolescence, although there are emotions for which the level of recognition is similar between the ages of 6 and 16, as in the case of joy, sadness, and anger (Lawrence et al., 2015).

Despite there being discrepancies in specific aspects of emotion recognition depending on the method used in the study, there is a consensus that children begin to identify four basic emotions at 3 years of age: joy, fear, sadness, and anger (Pons et al., 2004; Székely et al., 2011). Pons et al. proposed that, apart from recognizing emotions from facial expressions, children up to the age of 5 also begin to understand the causes of emotion. Later, and up to 7 years, children understand the mental nature of emotions and the possibility of hiding them. And in a third period, between 9 and 11 years of age, children understand ambivalence in emotions, moral emotions, and the cognitive regulation of emotions. The above study also indicated that understanding the external aspects of emotions is a prerequisite for understanding internal psychological aspects.

Previous research has shown that 6-year-olds can recognize some emotions - joy, sadness, and anger - in a similar manner to adolescents (Lawrence et al., 2015). But little (or less) is known about identifying these emotions in pretend situations. In this respect, in the present research two emotions with negative valence - sadness and anger - were selected to study the recognition of emotional expression in pretend play contexts. In addition, these two emotions are the first two negative emotions that children usually recognize (Widen, 2013), and may therefore also be the first ones to be interpreted in terms of pretend emotions. The emotion of happiness is usually the first to be labeled by children in free labeling tasks (Widen, 2013), but Sidera et al. (2011) pointed out methodological difficulties when studying children’s understanding of pretend happiness (children might interpret that pretending to be happy makes one actually happy), so we decided not to include pretend happiness in the present research.

Although many studies have been conducted on the recognition of basic emotional expressions in early childhood and in later development, few have focused on the recognition of pretend emotions. And despite this fact, emotions are often hidden or simulated for different reasons in everyday interpersonal communication and social relationships (see Zeman and Garber, 1996). Thus, the focus of this article is on this emotional simulation, and specifically, on pretend play situations where children express emotions that are different from their real ones for play purposes.

Children’s ability to understand that the real emotion of a person may differ from their emotional expression has often been studied in contexts of deception (see Sidera et al., 2013). Children begin to control their emotional expressions at the age of 4; however, at that age they are not yet able to deceive other people through emotional expression. This latter ability is closely related to the ability to understand that internal emotion and external emotion may differ, which usually develops between the ages of 4 and 6 (Harris et al., 1986; Pons et al., 2004; Misailidi, 2006; Sidera et al., 2012; Kromm et al., 2015).

Some studies have shown that children aged 8 to 12 may have difficulties in discriminating genuine from non-genuine emotional expressions (Dawel et al., 2015). The aforementioned authors found that children have particular difficulty with sadness, but not joy; adults have been found to be better at detecting both. In fact, at the age of 4 children are already able to explicitly discriminate between Duchenne vs. non-Duchenne smiles, and implicitly at the age of 3 (Song et al., 2016). That said, Dawel et al. (2015) considered that the skills required to carefully determine the authenticity of emotions from facial information mature at a later stage. There is evidence, then, that discriminating between genuine and pretend sadness is difficult in childhood, although we do not have enough information to know whether the same is true of anger (see Felleman et al., 1983).

Regarding research on emotional expression in contexts of play, the study by Mizokawa (2011), where children were presented with picture stories in a play context and in a non-play context, showed that 4- and 5-year-old were better at distinguishing pretend crying from real crying in a pretend play context than in a non-play context. So Mizokawa (2011) suggested that the context of pretend play facilitates children’s understanding of pretend crying. Furthermore, the study by Sidera et al. (2013) showed that at the age of 4, despite not mastering the distinction between internal and external emotion, most children understand the playful intentionality of emotions expressed in pretend play contexts. Specifically, Sidera et al. found that 4-year-old children are capable of understanding that when a character, or themselves, show a sad expression in a pretend play context, this person is just pretending, and is not really sad.

The present research aims to broaden the results of these studies by analyzing in greater depth the type of reasoning children use when it comes to detecting pretend emotions. This will allow us to identify which elements children who understand pretend emotions take into account that other children do not.

### Context and Emotional Expression Recognition

In daily life, faces are not usually seen in isolation. On the contrary, they appear in a multisensory context that includes aspects such as a voice, body posture and movement, or other people, and the recognition of facial expression is influenced by this context. Contextual influences are perceived early and automatically, and information provided by the facial expression is combined with that of the context (Righart and de Gelder, 2008).

The present study analyzes in greater depth children’s identification of and reasoning about simulated facial expressions with negative valence in a natural and playful context. We assume that this recognition incorporates contextual information in a natural and routine way, meaning that this is important for inferring the meaning of facial expressions. Hence, emotional perception is not only guided by the structural configuration of a person’s facial actions, but also from the context in which a face is encoded (Barrett et al., 2011), while we can also state that very young children appear to use contextual congruency, among other cues, to detect the authenticity of emotions (Walle and Campos, 2014).

Following Barrett et al. (2011), we consider that, although faces carry emotional information, their emotional meaning is constructed from the context in which they are embedded, and that people infer emotional meaning from facial movement and other social information (Barrett et al., 2019). In line with this, Keltner et al. (2019) hold that people’s interpretation of a target’s emotional expression is influenced by factors such as the following: who expresses the emotion (e.g., their gender); the mental states attributed to that person; the context (e.g., the action being undertaken by the person expressing the emotion); and the emotional expressions of the surrounding people.

Some studies with adults have shown the positive influence contextual information has on recognizing facial expressions (for a review, see de Gelder et al., 2006). There are also studies with children that have shown how a congruent visual context increases emotional recognition in children (Theurel et al., 2016), although other studies offer less clear results (Reichenbach and Masters, 1983; Nelson and Russell, 2011). Theurel et al. (2016) pointed out that there are methodological questions to consider here, and suggested that context may help to disambiguate the meaning of emotional expressions (e.g., sadness and fear, which it takes children a long time to discriminate between).

There is a discussion in this field regarding whether facial expression is the best clue for recognizing emotions in comparison to other sources of information (Face Superiority Effect), at least in early developmental stages (Denham, 1998). For example, Balconi and Carrera (2007) found that children recognize the emotions of joy and sadness better from facial expressions than from a story. However, they also found that the opposite is true with fear and disgust (Story Superiority Effect). These results suggest that the developmental order in which certain emotions are acquired is relevant when considering which informational sources are better for recognizing them. In line with this, the study by Nelson et al. (2013) also found that explaining a story provides a better clue for later emerging emotions than static or dynamic facial expressions (in this case, for the emotion of fear), whereas in children aged 3 to 5 and for the emotions of sadness and anger, still faces or videos were better than stories. To sum up, these results show that contextual information could be more important than facial expression in the recognition of emotions for complex emotions. This might also be the case for pretend emotions. In the cases of sadness and anger, the results published by Nelson et al. (2013) showed that children basically relied on still faces to detect anger, while for sadness they relied on videos showing different emotional cues (facial expression, voice, body posture, and movement).

In sum, despite prior research showing that some children aged 4 years are capable of understanding pretend sadness, some children are not. This previous research has not analyzed the reasoning children use when interpreting emotions expressed in pretend play contexts. Doing so would help to understand how children use contextual information in interpreting pretend emotions, and why some children understand emotions expressed in pretend play contexts as real. Moreover, prior research has studied how children understand pretend sadness, but we do not know whether children understand other negative emotions in a similar way. Therefore, as the labels for sadness and anger are the first labels for negative emotions that children acquire (Widen and Russell, 2003; Maassarani et al., 2014), we decided to study both the emotions of anger and sadness.

The aim of the present study, then, is to provide a more in-depth understanding of children’s recognition of and reasoning about pretend emotional expressions expressed in pretend play contexts. In this sense, we explored whether children’s capacity to detect pretend sadness and pretend anger vary by age or emotion. Moreover, to explore the role played by contextual information in identifying pretend emotions, we asked children to justify their interpretation of a pretend emotion in order to study what importance they award to information gleaned from facial expression as opposed to context. Specifically, our objectives are first, studying children’s recognition of simulated emotions in pretend play contexts for two emotions of negative valence; and second, exploring what kind of information children consider when performing this recognition.

## Materials and Methods

### Participants

The initial sample was comprised of 91 hearing children (46 girls and 45 boys) with a mean age of 72.74 months; *SD* = 18.86; range: 39 to 107 months. An initial emotion recognition task was administered in order to avoid difficulties with the pretend emotion understanding tasks. Thus, those children who failed to recognize the facial expressions of anger and sadness from the facial emotion recognition task were not included in the final sample of the study.

The final sample was comprised of 79 children (42 girls and 37 boys; mean age = 74.14 months; *SD* = 18.00; range = 40 to 107 months), who were separated into two age groups. Participants were divided into these two groups so as to detect developmental differences; the young group included children from preschool years, while the older group contained children from the first years of primary school. The group of young children was comprised of 36 children aged 3 to 5 (20 girls and 16 boys; mean age = 57.42 months; *SD* = 9.16; range = 40 to 71 months), and the older group contained 43 children aged 6 to 8 (22 girls and 21 boys; mean age = 88.14 months; *SD* = 9.56; range = 72 to 107 months). The Chi-Square test showed that there was not a significant relation between age group and gender (*p* > 0.05), so the sex distribution was similar between both age groups.

In Table 1, we describe some of the demographic information related to the children, separated by age groups.

**TABLE 1:** Mean values (and *SD*) in different variables related to the participants as a function of the age group.

	**Younger children**	**Older children**	**Age group comparison (Mann-Whitney)**
Number of siblings	0.79 (0.74)	1.15 (0.70)	*U* = 341.500 *Z* = −2.241 *p* < 0.05
Age of schooling	1.95 (1.02)	2.60 (0.81)	*U* = 129.500 *Z* = −2.333 *p* < 0.05
Level of studies of the father	1.95 (0.85)	2.00 (0.84)	*U* = 165.000 *Z* = −0.193 *p* > 0.05
Level of studies of the mother	1.95 (0.74)	2.20 (0.83)	*U* = 172.500 *Z* = −1.041 *p* > 0.05

*The variable level of studies had 4 categories: 0 = does not have studies; 1 = primary education level; 2 = secondary education level; 3 = higher studies.*

In the area where the study was conducted, the main school languages were Catalan and Spanish, so all the selected children knew at least one of the two languages, and all families but three informed us that at least one of the parents communicated to their child either in Catalan or Spanish.

The children were recruited from four state-run schools in Spain. Written informed consent was obtained from parents before administering the tasks to their children. None of the children were reported to have cognitive delays.

### Materials

Five experimental tasks were administered to the children, and their teachers were asked to complete two questionnaires. However, of the five experimental tasks, only the following three tasks are considered in the present study (thus, the results of an expressive vocabulary task and a pretend actions task are not considered here):

(a) Facial emotion recognition task. A task of facial emotion recognition (FER) was included to ensure children did not fail the pretend emotions task due to difficulties recognizing the emotions of anger and sadness. The FER task included six drawings of a girl showing six basic emotions (happy, sad, scared, angry, surprised, and disgusted; the drawings for the task are included in Sidera et al., 2017). The six drawings were placed in two lines in front of the child, and the experimenter labeled the emotions one by one (following a Latin-square design to counterbalance the order of presentation). After identifying a label for one emotion, the experimenter asked the child “*Could you point to the girl looking…?”* After this question, the experimenter said “OK” and proceeded to label the following emotion. In the present study, only the results of the emotions of anger and sadness were considered. Children who pointed correctly to the faces of anger and sadness were included in the final sample of the study, whereas those who failed at least one of these emotions were excluded.
(b) Pretend emotions task. A task with silent videos (lasting about one minute each) was used to evaluate children’s reasoning that the emotions used in pretend play contexts may be expressed with playful purposes. This task consisted of a warm-up phase and a test phase. In the warm-up, children were again shown the drawings of sadness and anger from the FER task, and were asked about the emotion expressed by the girl in each of the two drawings: “Can you tell me how this girl feels”? Children who responded incorrectly were given corrective feedback (the correct label was stated). Moreover, in order to make children familiar with the words that the present study used to refer to the distinction between pretense and reality (we used two Catalan expressions for making this difference: “de veritat” and “de mentida”), the experimenter performed some real actions and some pretend actions. First of all, the experimenter did two actions without feedback, and then four more actions with feedback. The first action without feedback was the real action of drinking water. Before doing the action, the experimenter explained it to the participant: “Now I’m going to do a real action, ok? I will really drink water.” Then, the experimenter drank some water from a glass, and said: “Did you see? I really drank water.” The second action without feedback was pretending to drink water. The experimenter also explained the action beforehand (“Now I am going to do a pretend action. I will pretend to drink water”). The pretend action was carried out in an obvious pretend way (the glass was empty, the lips did not touch the glass, and the movements were exaggerated as it is usual in pretend play), and after the action the experimenter said: “Did you see? I pretended to drink water. I pretended to drink, but in reality I did not drink.” Afterwards, the experimenter carried out the four actions with feedback. Before carrying out each action, the experimenter said: “Ok, X, now I am going to do an action and you have to tell me whether it is a real or a pretend action, ok?” After that, the experimenter carried out the action and asked whether it was real or pretend. For example: “X, am I really cutting the paper or am I pretending to cut the paper?” After their response, children were given corrective feedback. For example: “Yes, very good, I pretended to cut the paper with the scissors, but I did not really cut it,” or “Really cutting the paper? No, I pretended to cut the paper with the scissors, but I did not really cut it.”

In the test phase, eight silent videos were presented of children acting out real or pretend emotions (four videos of real emotions and four of pretend emotions) following a Latin-square design to counterbalance the order of presentation. However, in the present study we were interested in how children reason about emotions expressed in pretend play contexts, so only the four videos depicting pretend emotions were analyzed. In these videos, two characters were practicing pretend play and at the end the image froze with one of the characters (the “protagonist”) expressing pretend anger or pretend sadness (two videos of each emotion were used). In the pretend sadness videos, one character played the role of the baby and the other the role of the mother; the mother became angry after the baby misbehaved (did not want to eat or sit down in a chair), so the baby pretended to be sad. In the pretend anger videos, two children pretended that a doll was misbehaving (throwing pretend food or knocking down a tower of blocks) and one of them pretended to be angry toward the doll.

At the end of each video, while the image was frozen, two questions were asked about the protagonists:

Test question: *“Is the child really angry/sad or is she pretending to be angry/sad?”*

Justification: *“Why do you think she is angry/sad (or pretending to be angry/sad)?”*

(The word “angry” was used for the pretend anger videos and the word “sad” for the pretend sadness videos).

Hence, the test question evaluated whether children understood the expressed emotion as real or pretense, and thus that emotions may have a pretend purpose. One point was given for each correct answer in the test questions, so the total score for the pretend emotions task ranged from 0 to 4. Regarding justifications, they were divided into the following categories:

1. *Emotion*. When children justified their response to the test question with reference to the emotional expression or the emotion of the protagonist.
2. *Event/behavior.* When children justified their response to the test question by referring to the event in the video that triggered the protagonist’s emotion (e.g., *“because the doll knocked down the tower”*), or when they referred to the protagonist’s behavior (*“because the girl is telling the doll off”*).
3. *Play.* When children justified their response to the test question by arguing that the protagonist was playing (e.g., *“they were just pretending with the doll*”) or explaining that the children were just pretending so the emotion of the protagonist must be understood as pretense (e.g., *“because it was the girl who knocked down the tower, not the doll”).*
4. *Non-response.* When children did not answer, said they did not know the answer, or gave a non-sensical answer.
5. *Other.* Answers that included more than one of the previous categories were included in this category.

Two authors of the study categorized all responses into one of the five previous categories, and their categorizations were compared. The number of observed agreements was 91.46% of the observations, while the Kappa equaled 0.884 (*SE* = 0.021). Differences between judges were resolved by discussion.

Finally, the categories event/behavior and play were merged in some analyses, as both include information related to the context of the story represented in the videos.

(c) Non-verbal cognitive ability test. The children’s non-verbal ability was evaluated by means of the Pattern Construction subtest from the British Ability Scales, 2nd edition (Spanish version by Arribas and Corral, 2011). The Ability Scores of the test were used, as they consider the specific items administered to each child.

Aside from the tasks administered to the children, their teachers were also asked to respond to a language assessment questionnaire [the Language Proficiency Profile LPP-2 by Bebko and McKinnon (1993)], the data from which were not used in the present study. The teachers also responded to a demographic questionnaire in order to provide background information about the children (date of birth, number of siblings, school enrollment, existence of learning difficulties, parental education, mother tongue of the parents, and language used with the child).

### Procedure

The children were tested in a quiet room in their schools. Administration of the tasks lasted between 35 and 55 min and took place in one session. The data were analyzed using IBM SPSS version 23. Non-parametric tests were used, as the data did not meet the criteria of normal distributions. The one-sample Wilcoxon signed-rank test was used to compare children’s scores to chance level. The Mann-Whitney’s *U* test was used to compare the scores between the two age groups. The Wilcoxon test was used to compare the scores of the pretend sadness with the pretend anger videos. Finally, the Chi-Square test was used to compare frequencies of responses between the different justification categories.

## Results

### Scores for the Pretend Emotions Task

The younger children’s mean in the pretend emotions task was 2.5 (out of 4; *SD* = 1.30) and the older children’s mean was 3.84 (*SD* = 0.37), close to the maximum. Children’s scores in each age group were compared to chance expectation (two points was considered as the chance level, because the task involved four dichotomous responses) using the one-sample Wilcoxon signed-rank test. Both groups obtained scores above chance (young group: *Z* = 2.043, *p* = 0.041; older group: *Z* = 6.168, *p* < 0.001). On the other hand, Mann-Whitney’s *U* test showed significant differences between the two age groups (*U* = 281.500; *Z* = −0.5447; *p* < 0.001). The mean percentile in the non-verbal cognitive ability score was 60.42 (*SD* = 25.15) in the young group and 63.84 (*SD* = 20.55) in the older group, and according to Mann-Whitney’s *U* test no significant differences existed between the two age groups in terms of the percentile of non-verbal ability (*p* > 0.05).

When type of emotion was taken into account, age differences were observed for both sadness and anger, the older children doing better than the younger children. Moreover, both the younger and older children obtained better scores in the pretend sadness videos than in the pretend anger videos (see Table 2). When we compared children’s scores for each type of emotion at each age group to the expected chance level (1 point), we observed that older children obtained scores above chance in both emotions (anger: *Z* = 6.000, *p* = 0.000; sadness: *Z* = 6.557, *p* < 0.001), while young children scored above chance for sadness (*Z* = 3.889, *p* < 0.001) but not for anger (*p* = 0.414).

**TABLE 2:** Means (and SD) for the pretend emotions task by age and type of emotion.

	**Anger**	**Sadness**	**Anger-sadness comparison (Wilcoxon)**
Younger children	0.89 (0.82)	1.61 (0.73)	*Z* = −3.802 *p* < 0.001
Older children	1.84 (0.37)	2 (0)	*Z* = −2.646 *p* = 0.008
Age group comparison (Mann-Whitney)	*U* = 292.000 *Z* = −5.361 *p* < 0.001	*U* = 580.000 *Z* = 1246.500 *p* = 0.001	

*score range 0–2.*

**FIGURE 1:**
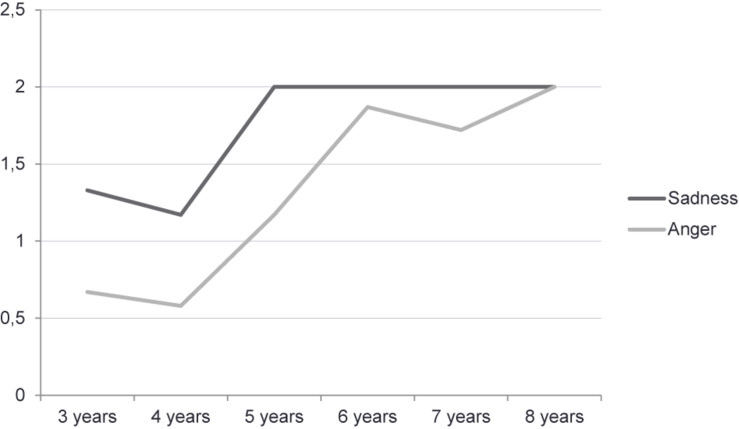
Developmental understanding of pretend sadness and pretend anger as a function of age.

On the other hand, the development of the recognition of pretend sadness and pretend anger is shown in Figure 1. The same pattern of development is observed, but with a better performance for the emotion of sadness than for that of anger. Specifically, children reached the maximum score for sadness at the age of 5, and a near-to-ceiling score for anger at the age of 6.

### Justifications for Responses in the Pretend Emotions Task

Regarding the mean number of each type of justification used by the children (see Table 3), we observed that in young children the most used category was event/behavior, while older children mostly used the categories emotion and play. Furthermore, age differences were found in how the children justified pretend emotions: among the older children, there was a significantly higher use of the emotion and play categories and lower use of the event/behavior category. Also, the decrease with age in the number of justifications in the non-response category was close to significant.

**TABLE 3:** Mean number (and SD) for justifications used in the four scenarios of the pretend emotions task.

	**Emotion**	**Event/behavior**	**Play**	**Non-response**
Younger children	0.75 (1.08)	1.81 (1.37)	0.75 (1.08)	0.69 (1.17)
Older children	1.33 (1.25)	0.91 (1.13)	1.40 (1.07)	0.35 (0.923)
Age group comparison (Mann-Whitney)	*U* = 5611.500 *Z* = −2.221 *p* = 0.026	*U* = 486.000 *Z* = −2.990 *p* = 0.003	*U* = 485.000 *Z* = −2.987 *p* = 0.003	*U* = 625.000 *Z* = −1.917 *p* = 0.055

*As there were four videos, the maximum number of justifications for each category was 4.*

Following this, we analyzed the type of justification as a function of the emotion involved in the videos (anger vs. sadness; see Table 4). The children were found to use different types of justification as a function of emotion type. In the anger videos, children mostly used play and event/behavior justifications, while in the sadness videos the most commonly used justifications were emotion and event/behavior. In fact, the category emotion was used significantly more in the sadness than in the anger videos, while the children more frequently used the play category for the anger videos than for the sadness videos.

**TABLE 4:** Mean number (and SD) for justifications used in the pretend emotions task as a function of emotion type.

	**Emotion**	**Event/behavior**	**Play**	**Non-response**
Anger videos	0.23 (0.53)	0.73 (0.83)	0.85 (0.83)	0.19 (0.51)
Sadness videos	0.84 (0.87)	0.56 (0.75)	0.28 (0.58)	0.32 (0.67)
Anger-sadness comparison (Wilcoxon)	*Z* = −5.202 *p* < 0.001	*Z* = −1.737 *p* = 0.082	*Z* = −4.739 *p* < 0.001	*Z* = −1.978 *p* = 0.048

*As there were two videos for each type of emotion, the maximum number of justifications for each category was 2.*

We also analyzed the use of the different categories in the anger and sadness situations as a function of the age group (see Table 5). For the emotion category both the young and the older group followed the same pattern: children used this type of justification more in the sadness than in the anger situation. For the event/behavior category, the young group showed a higher use of this category in the anger situations, and no differences existed in the older group. The opposite occurred in the play category: the older group showed a higher use in the anger situation than in the sadness situation. Finally, the older group showed a higher use of the non-response category in the sadness situation, but no differences existed in the young group.

**TABLE 5:** Mean number (and SD) for justifications used in the pretend emotions task as a function of emotion type and age group.

		**Emotion**	**Event/behavior**	**Play**	**Non-response**
**Young group**	Anger	0.17 (0.45)	1.08 (0.87)	0.44 (0.74)	0.31 (0.62)
	Sadness	0.61 (0.77)	0.72 (0.78)	0.28 (0.62)	0.39 (0.73)
Anger-sadness comparison (Wilcoxon)		*Z* = −3.234 *p* = 0.001	*Z* = −2.166 *p* = 0.030	*Z* = −1.261 *p* = *0.207*	*Z* = −0.758 *p* = 0.448
**Old group**	Anger	0.28 (0.59)	0.44 (0.67)	1.19 (0.76)	0.09 (0.37)
	Sadness	1.02 (0.91)	0.42 (0.70)	0.28 (0.55)	0.26 (0.62)
Anger-sadness comparison (Wilcoxon)		*Z* = −4.122 *p* < *0.001*	*Z* = −0.179 *p* = *0.858*	*Z* = −4.786 *p* < *0.001*	*Z* = −2.333 *p* = *0.020*

In order to evaluate the relationship between each justification category and correct responses to the test questions for the pretend emotions task, the proportion of correct responses was calculated for each justification category. The proportion of justifications labeled as play and considered correct was 0.98. This proportion was 0.89 for the emotion category, 0.70 for the non-response category, and 0.63 for the event/behavior category. A Chi-Square test revealed significant differences in the proportion of correct responses between categories (χ*^2^* = 44.601, *p* < 0.001). When the proportions of correct responses were compared between the different categories in pairs, significant differences were observed between the following categories: emotion and event/behavior (χ*^2^* = 16.845, *p* < 0.001), emotion and play (χ*^2^* = 5.390, *p* = 0.020), emotion and non-response (χ*^2^* = 6.996, *p* = 0.008), play and event/behavior (χ*^2^* = 35.578, *p* = 0.000), and play and non-response (χ*^2^* = 22.236, *p* < 0.001). No differences were found between the categories event/behavior and non-response (*p* > 0.05).

Proportional use of the emotion and contextual categories (the latter including the categories event/behavior and play) was compared in children who gave correct responses. The children responded with a contextual category in 0.59 of correct responses; with an emotion category in 0.29 of cases; with a non-response category in 0.11 of cases; and in the other category in 0.004 of cases. Therefore, the contextual category was the most widely used category for correct responses. However, when the variable type of emotion was taken into account, a higher use of the contextual category over the emotion category (for correct responses) was noted for the anger videos, but not for the sadness videos (see Table 6). Thus, for the sadness videos, the proportion of correct responses was very similar in the contextual and emotion categories. When the Chi-Square test was used to compare the proportion of correct responses for emotion vs. contextual in the anger and sadness videos, significant differences were observed between the two conditions in the use of the two categories (χ*^2^* = 38.234, *p* < 0.001).

**TABLE 6:** Proportional use of the different justifications in the pretend emotions task as a function of type of emotion and age group.

		**Emotion**	**Contextual**	**Non-response**	**Others**
All children	Anger videos	0.11	0.82	0.07	
	Sadness Videos	0.43	0.42	0.14	0.01
Young group	Anger videos	0.03	0.84	0.13	0
	Sadness Videos	0.31	0.53	0.16	0
Old group	Anger videos	0.14	0.81	0.05	0
	Sadness Videos	0.51	0.35	0.13	0.01

Finally, the proportional use of the emotion and contextual categories (in children who gave correct responses) was compared as a function of the age group for each emotion (see Table 6). In the young group, the contextual category was the most used both for the anger and sadness videos. The older group also used the contextual category more than the emotion category for the anger videos, but they used the emotion category more for the sadness videos. The Chi-Square confirmed that the use of the emotion and contextual categories was similar in the young and older groups for the anger videos (*p* > 0.05), while the age groups differed in the frequency of the use of these categories for the sadness videos (χ*^2^* = 6.028, *p* = 0.014).

## Discussion

Previous literature has shown that young children are capable of realizing other children may pretend to be sad for playful reasons (see Sidera et al., 2013). The current study found that this knowledge develops gradually between the ages of 4 and 6, and is well-established from the age of 6 years, as all children in the older group recognized the expression of pretend sadness. This older group also performed well at the pretend anger task, although significant differences in their understanding of pretend anger and pretend sadness existed. Furthermore, younger children (aged 3 to 5) performed worse than older children, especially in the pretend anger task. So while the young group showed some understanding that sadness may be expressed for playful intentions, their scores in the pretend anger tasks were not above chance level. More research is needed to confirm the possibility that children’s capacity to interpret pretend sadness is better than their capacity to interpret pretend anger, and to discard the possibility that methodological differences between the two tasks accounted for the differences we found.

The results of this study suggest that recognizing negative pretend emotions (for anger and sadness) is easier than discriminating genuine from non-genuine expressions. This statement is supported if we compare our results to those of Dawel et al. (2015); while they found that compared to adults, children aged 8–12 had difficulties discriminating genuine sadness, in our study even young children recognized expressions of pretend sadness quite well. In sum, we can state that being able to recognize facial expressions is not enough for recognizing pretend emotions (let us recall that all children in our study had successfully completed a task of recognizing the emotions of sadness and anger, but some failed the pretend emotions task); that said, children are capable of identifying pretend sadness before they are capable of distinguishing genuine from non-genuine facial expressions of sadness out of context (as in Dawel et al. study); hence, it is likely that children use other informational cues to identify pretend emotions. Indeed, the capacity to use contextual information to detect authenticity in emotional expressions appears very early. Walle and Campos (2014) showed that 19-month-olds are capable of detecting authenticity in emotional expressions based on contextual information (expressing pain in a situation where a hammer did not hit the hand).

In this study, we grouped the types of reasoning children use to justify whether an emotion is pretend or not into three categories: referring to the protagonist’s emotional expression (emotion), considering the context of pretend play (play), or referring to the context of the story depicted in the video and/or the behavior of its protagonists (event/behavior). The results showed differences between the age groups, since the younger children (3- to 5-year-olds) tended to justify the pretend emotions expressed by the protagonists of the story by referring to their context or the behavior of their characters, while the older children (6- to 8-year-olds) mostly referred to the protagonist’s emotional expression and the context of pretend play. Furthermore, the justifications emotion and play, which were used more frequently by the older children, were the ones most associated with correct responses. Therefore, we can state that there are developmental differences in how children explain whether an expressed emotion is pretend or real: as children grow older they do not consider the general context of the story as much, but rather focus more on the fact that the depicted story is set in a pretend play situation; similarly, they do not focus much on the general behavior of the protagonist, but specifically on their emotional expression. These results are in accordance with those found by Sidera (2009), whose study involved children being told stories where the protagonists simulated sadness or happiness in a pretend play context. When they were asked to justify the external and internal emotion of the protagonist, 6-year-olds were more capable of considering that the protagonists were involved in a playful situation than 4-year-olds.

In our study, we observed differences in the reasoning children used according to the type of pretend emotion expressed in the videos. For anger, most children referred to the play context or to the event described in the story or the behavior of the protagonist rather than the protagonist’s emotional expression. This was true for both age groups, although older children more frequently used the play category, in accordance with the developmental differences commented above. For sadness, children’s justifications were mostly based on the emotional expression of the protagonist. When age groups were considered we found that young children mostly used the event/behavior category, while older children mostly used the emotion category. Before we discuss a possible explanation of these differences, it is worth mentioning some of the justifications given by the children. We need to consider that a proportion of the children did not justify their response, and also that the least successful justification referred to the event and/or the behavior of the protagonist. This is possibly because the latter involves considering elements of context or behavior (beyond those related to playing) that are less relevant for interpreting facial expression correctly.

Success in the event/behavior category was near chance level. Therefore, the behavior or situation/event in which the emotional expression is integrated would not be useful in this situation for detecting pretend emotions, while knowledge of the general context of play in which the emotion is simulated would be. Therefore, as found in other studies (Balconi and Carrera, 2007; Nelson et al., 2013; Widen et al., 2015) with regard to later emerging emotions, prior history or, in this case, viewing the emotional expression to be identified in a story (where children play), facilitates recognition that the emotional expression is a pretend one. Children’s references to the protagonist’s emotional expression were also associated with correct responses, possibly because this is linked to children’s capacity to capture the exaggerated elements of the facial expression. Although we cannot conclude this from the data in our study, the study by Walle and Campos (2014) does support the view that infants as young as 19 months of age are sensitive to exaggerated emotional displays and may use the level of exaggeration of an emotion in order to judge its authenticity or communicative value. Interestingly, in our study older children used mostly the emotion category for justifying pretend sadness, while they mostly used the play category for justifying pretend anger. We will try to interpret this next, by looking at the categories used for the correct responses.

Finally, when the results of the correct answers were only grouped into two categories (contextual vs. emotion), it was found that in the case of anger, children mostly used contextual clues (and not emotion) to judge whether the emotion was pretend or not (both in the young and the older group). In the case of sadness, children used both categories similarly when the whole sample was taken into account. But when age groups were considered, we found that young children’s interpretations were more based on contextual cues while older children used emotion cues. Gnepp (1983) found that even preschoolers were capable of considering both emotional and contextual cues when presented with pictures where the facial expression of the protagonist was incongruent to the context. In this sense, the age changes in the justifications for pretend emotions would not be attributable to the inability of young children considering one or another type of cue. In this sense, a possible explanation for our results is that it is easier to detect (or express) pretend sadness than pretend anger from facial cues; this would explain why children relied more on the expressed emotion in the pretend sadness videos (since it was enough for children, especially for the ones in the older group, to interpret the communicative intention of the protagonist), whereas in the pretend anger videos children needed to seek more contextual cues (and especially cues related to the play situation in the older group) to interpret the pretend emotion and give an answer. Future research should clarify whether this explanation is correct, or whether differences are due to methodological issues.

This study had some limitations. First, silent videos were used to control for the influence of information from the intonation of speech, although obviously there is normally sound and language when we are exposed to the emotional expressions of others. Research into the recognition of emotional expressions by adults has shown that this is modulated by linguistic stimuli, and it is therefore necessary to advance the recognition of pretend emotions through more ecologically valid situations (Park and Itakura, 2019), which include the information provided from the prosody that accompanies speech as well as from some vocal bursts. Moreover, there is evidence that anger and sadness may be differentiated from the expression of other emotions in different modalities (Keltner et al., 2019), but it is yet to be investigated whether this is the case for pretend emotions. Similarly, the level of the intensity of the emotions from the facial emotion recognition task or from the pretend emotions task were not controlled. We must also bear in mind that in the present study children were asked to justify whether the emotions expressed by other children were pretense or not, meaning they were asked to give explicit responses, whereas if implicit behaviors were sought, then different, and perhaps interesting, results may also be obtained. Furthermore, the study of emotional expressions suggests that they are expressed in prototypical multimodal patterns of behavior with important variations (Keltner et al., 2019), a theory that also needs to be investigated for pretend emotions.

To sum up, then, in this study we have found that children aged 3 to 5 are capable of detecting pretend sadness in other children, at least in a contextualized situation, but still have difficulties with pretend anger. This may be due to the fact that facial cues are not as evident for pretend anger, and they have to seek more contextual cues. When doing so, older children are more aware when a character’s behavior should be interpreted as pretend play, and therefore also interpret their emotional expressions in these terms.

## Data Availability Statement

The raw data supporting the conclusions of this article will be made available by the authors, without undue reservation.

## Ethics Statement

The studies involving human participants were reviewed and approved by Comité d’ètica i bioseguretat de la recerca de la Universitat de Girona. Reference: CEBRU0024-2019. Written informed consent to participate in this study was provided by the participants’ legal guardian/next of kin.

## Author Contributions

All authors contributed to the study conception and design, commented on previous versions of the manuscript, read and approved the final manuscript. FS, ES, and AA prepared the material and collected and analyzed the data. ES and FS wrote the first draft of the manuscript.

## Conflict of Interest

The authors declare that the research was conducted in the absence of any commercial or financial relationships that could be construed as a potential conflict of interest.
